# A Data-Driven Framework for Class Overlap Reduction to Improve Soft Sensor Performance

**DOI:** 10.3390/s26154826

**Published:** 2026-07-30

**Authors:** Faizal Widya Nugraha, Thanda Shwe, Israel Mendonça, Masayoshi Aritsugi

**Affiliations:** 1 Graduate School of Science and Technology, Kumamoto University, Kumamoto 860-8555, Japan; 2 Faculty of Advanced Science and Technology, Kumamoto University, Kumamoto 860-8555, Japan; 3 Research and Education Institute for Semiconductors and Informatics, Kumamoto University, Kumamoto 860-8555, Japan

**Keywords:** data-driven, machine learning, resampling methods, class imbalance, class overlap, service industry

## Abstract

In the data-driven applications, class overlap and class imbalance are major factors that degrade machine learning performance. While existing resampling methods often address these issues independently, they frequently fail to preserve the underlying distribution of the majority class after cleaning. This study proposes Two-Phase Clustering Plus, a novel framework that uniquely leverages overlap detection to inform the undersampling process. Unlike conventional approaches that remove samples indiscriminately, this framework integrates the Edited Nearest Neighbors technique to isolate overlapping regions, applying data cleaning strictly to the majority class. To manage the trade-off between overlap reduction and the preservation of informative boundary samples, this localized cleaning strategy is combined with a two-phase clustering strategy that retains the safe majority data. This synergy ensures the targeted elimination of ambiguous majority instances without causing excessive information loss, systematically selecting representative samples that maintain critical boundaries and the global data structure. Evaluated on five service industry datasets representing virtual sensing environments derived from customer behavioral data, experimental results demonstrate that the proposed method yields a 1.45% improvement in geometric mean and significantly enhances class separability. By explicitly linking overlap removal with strategic clustering, this approach provides a robust and distribution-aware solution for complex imbalanced datasets.

## 1. Introduction

In the increasingly digitalized modern industrial landscape, sensor-based systems play a crucial role in collecting measurement data to support performance monitoring, analysis, and decision-making. The development of the Internet of Things (IoT), data acquisition systems, and computing infrastructure has enabled various types of physical and virtual sensors to generate large volumes of data at high frequencies. The primary challenge is no longer simply data availability but how to accurately and efficiently process valuable information from sensor data. In this context, machine learning (ML) is a key component that enables modeling complex relationships among measurement variables and supports advanced core functions such as anomaly detection, monitoring, and prediction [1,2].

As systems become increasingly complex, achieving comprehensive data acquisition through physical instrumentation often faces challenges in terms of economic viability and measurement precision [3]. To enhance measurement precision and cost-effectiveness, the concept of soft sensors (SS) has been developed. These are virtual sensors that estimate target variables using available measurement data and mathematical models or machine learning. Whereas physical sensors typically rely on hardware, SSs operate at the computational layer and extend core capabilities through data-driven inference [2,4].

Over time, SSs have evolved beyond simple prediction tools and have become an integral component of intelligent core architectures. By improving ML, SSs are now able to capture nonlinear relationships among measured variables, accommodate complex system dynamics, and enhance the quality of sensing information without increasing hardware costs [2].

Because SSs have been extensively applied in manufacturing systems and industrial processes, their adoption in service industries such as telecommunications, banking, retail, and other digital services presents an opportunity for technology development. In these domains, although physical sensors may not be directly involved, customer activities continuously generate data that can be treated as virtual measurements. Transaction records, service usage data, customer interactions, and system activity logs serve as historical data that indirectly represent customer states and behaviors [5,6,7].

In this context, machine learning-based soft sensors (ML-SSs) serve as inferential mechanisms for estimating latent customer conditions, such as satisfaction levels, loyalty, or the likelihood of service discontinuation (customer churn) [8,9]. By utilizing readily available behavioral data, SSs enable continuous monitoring of customer states without requiring additional physical measurement instruments. Accordingly, customer churn prediction can be viewed not only as a classification problem but also as a form of state sensing that captures the dynamics of customer behavior in service systems.

Although ML-SS offers high flexibility and inference capabilities, its performance is highly dependent on the quality of the sensing data used. One of the most common problems in the development of ML, particularly in service industry applications, is class imbalance (CI), where the number of samples in the majority class is significantly greater than that in the minority class. This imbalance can bias the classifiers towards the majority class, limiting its ability to detect minority conditions [10,11].

In addition to CI, another problem that often occurs in parallel is class overlap (CO), where conditions from different classes overlap in the feature space [12]. From a ML perspective, CO reflects measurement uncertainty or an ambiguous region, where the characteristics of two distinct conditions are difficult to distinguish. The presence of CO degrades ML performance by blurring the decision boundary and increasing the risk of inference errors [13].

Therefore, addressing CI without considering CO often fails to provide optimal performance improvements. To improve overall quality, an approach is needed to simultaneously balance the data distribution and reduce ambiguous measurement regions [13], thereby ensuring more reliable classification outcomes across various data distributions.

Numerous resampling strategies have been proposed to improve the performance of ML-SS, particularly for handling CI. Traditional resampling techniques generally fall into two categories. Undersampling approaches balance the classes by removing data from the majority class. However, these methods have a major drawback: the potential loss of informative boundary samples caused by majority-sample removal [14]. Conversely, oversampling techniques create synthetic data for the minority class, which increases the risk of overfitting [15].

Recently, the Two-Phase Clustering (TPC) method has emerged as a reliable option for handling class imbalance [16,17]. However, TPC still has several limitations. First, its extensive removal of majority class data can inadvertently discard informative samples. Second, its reliance on linear programming in the first phase creates high computational complexity, limiting its scalability on large datasets. Furthermore, in scenarios with significant CO, its performance can be constrained by the geometric rigidity of the Convex Hull Clustering (CHC) mechanism [18]. This rigid boundary formation acts as a broad envelope that encompasses ambiguous regions where both classes coexist. Consequently, the formed clusters fail to effectively separate the majority class from the overlapping zones, which reduces the overall robustness and classification performance.

To address this limitation, this study proposes the Two-Phase Clustering Plus (TPC+) framework. Unlike standard TPC, the proposed approach introduces a topological cleaning stage based on the Edited Nearest Neighbors (ENN) principle [19]. By removing locally inconsistent samples near class boundaries prior to clustering, this step reduces local ambiguity in overlapping regions and mitigates the geometric sensitivity of CHC. As a result, the clustering process operates on a more structured data space, facilitating more stable cluster formation and a more reliable decision boundary. Consequently, the sampling process can select more representative and informative samples from each cluster.

This design is aligned with prior studies that highlight the importance of improving data quality and structural initialization in clustering-based ML. For example, ref. [20] employs density-based clustering techniques such as DBSCAN to isolate anomalous patterns and reduce the influence of irregular data points. In a different context, ref. [21] integrate K-Means clustering with Hidden Markov Model, where clustering serves as a data-driven initialization that improves model stability and convergence. Inspired by these perspectives, the proposed framework integrates boundary-aware preprocessing with structure-aware initialization, thereby enhancing the robustness and effectiveness of clustering-based learning.

The proposed approach is evaluated on multiple service industry datasets, including the telecommunications, banking, retail, and travel sectors. These datasets represent virtual sensing environments derived from customer behavioral data. Experimental results show that integrating CO reduction before data balancing improves training data quality. This integration ultimately enhances the performance of ML-SS.

The main contributions of this study are summarized as follows:

- We propose a resampling framework that integrates TPC with an overlap-reduction mechanism to eliminate ambiguous majority samples before addressing class imbalance.
- We conducted extensive evaluations using multiple sampling methods, classifiers, and diverse datasets across domains to assess the robustness of the proposed methods.

The rest of this paper is structured as follows: Section 2 reviews the related work. Section 3 introduces the proposed methodology. Section 4 details the experimental setup. Section 5 presents and discusses the experimental results. Section 6 limitations and future directions. Finally, Section 7 provides the conclusions of this study.

## 2. Related Work

### 2.1. Soft Sensors

Soft sensors are virtual systems used to estimate variables that cannot be directly measured, known as latent states, by processing historical data. At its core, the logic of reconstructing hidden information from observable data can be applied far beyond industrial settings [1,2]. In the service industry, this framework can be reconceptualized as a behavioral soft sensor. In this context, customer churn is treated as a virtual sensor reading inferred from behavioral proxies like transaction history and interaction patterns. This unified perspective highlights that effective prediction, whether in a market, depends on accurately capturing latent dynamics. By linking observed data to actionable insights, this framework provides a solid basis for applying advanced industrial methodologies to the complexities of human behavior.

### 2.2. Handling Class Imbalance and Class Overlap

The imbalanced data problems and overlap are persistent challenges in predictive modeling, often biasing algorithms toward the majority class and degrading performance on minority instances [14]. Traditional data-level approaches, such as Random Undersampling (RUS) and Random Oversampling (ROS), attempt to address imbalance but may discard crucial structural information or induce overfitting [15,16,22]. Boundary-cleaning techniques, including Tomek Links (TL) and Neighborhood Cleaning Rule (NCR), target overlapping instances but are often either too conservative or too aggressive [23,24], especially in highly imbalanced datasets. Hybrid methods, such as KMSMOTE [25], provide more sophisticated solutions but may remain sensitive to parameter choices. CTGAN [26] generates samples within overlapping regions, blurring decision boundaries rather than clarifying them.

These limitations reveal a critical conceptual gap, most existing methods address either imbalance or overlap, but rarely integrate both while preserving structural separability, which is essential for maintaining clear decision boundaries in high-dimensional spaces [27,28]. In this context, the role of ENN as a specialized overlap reduction filter has been highlighted in recent literature. Studies by [13,29] demonstrate that incorporating ENN into clustering or generative frameworks enhances the clarity of decision and mitigates the blurring effect caused by noisy majority or synthetic instances. Building upon these insights, there is a need for approaches that combine overlap reduction and structural alignment to improve the reliability of cluster-based resampling. This conceptual motivation underpins the development of TPC+, which operationalizes these principles by systematically addressing CO and structural distortion prior to clustering. By emphasizing distribution-aware and structure-preserving strategies, TPC+ provides a robust foundation for minority class classification, bridging the gap between distributional balancing and structural integrity.

## 3. Methodology

### 3.1. Introduction of Proposed Framework

**Figure 1:**
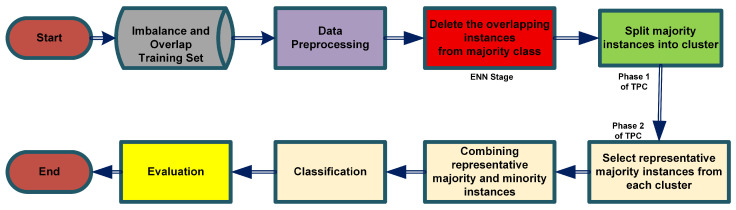
Workflow of Proposed Framework.

We introduce the proposed Two-Phase Clustering Plus (TPC+) method, designed to improve the handling of CO in imbalanced datasets. This method enhances the traditional TPC by addressing challenges associated with overlapping classes. CO occurs when feature attributes lack sufficient discriminative power, causing samples from different classes to occupy the same geometric regions in the feature space. This phenomenon is typically driven by intrinsic data ambiguity or attribute noise, which obscures the underlying separation boundaries. Consequently, the theoretical misclassification risk increases, making it difficult for classifiers to establish an optimal decision boundary and leading to a predictive bias toward the majority class. The TPC+ framework systematically addresses this by using ENN as a filter to reduce overlap density in boundary regions, while TPC organizes the remaining data into more homogeneous sub-clusters to significantly enhance class separability. The comprehensive workflow of our framework is shown in Figure 1.

### 3.2. Class Overlap Reduction

In this section, we explain the CO reduction of TPC+. Minimizing class overlap enhances separability, which in turn enables the classifier to achieve better performance. The ENN method is an undersampling technique that can be used to clean overlapping regions in imbalanced datasets. Its mechanism is based on the fundamental concept of k-Nearest Neighbors, which assumes that each instance tends to share the same class as its closest neighbors [19]. ENN operates under the principle of data cleaning, removing instances that are inconsistent with their surrounding neighborhood. The procedure can be summarized as follows: For each instance in the dataset:

- Identify its *k* nearest neighbor.
- Determine the majority class label among those neighbors.
- If the label differs from the majority of its neighbors, the instance is considered noise or a borderline sample and is removed from the dataset.
- If the label matches the majority of its neighbors, the instance is retained.

### 3.3. Two Phase Clustering Based Undersampling for Handling Class Imbalanced

In this section, we explain how we integrated TPC into our framework. The study conducted by [16,17] was specifically designed to address the CI problem. Although the TPC method has shown effectiveness in handling class imbalance problems, its performance can still be limited by the rigid geometric structure mechanism [18]. In datasets with high class overlap, this rigid boundary may not adapt well to ambiguous regions where majority and minority samples are mixed together. As a result, important boundary regions may not be properly represented, which can reduce classification performance. However, we expect that the proposed class overlap reduction component can help overcome these limitations by improving class separability in overlapping regions. TPC is divided into two main phases. The details are as follows:

### 3.4. Phase 1: Clustering Phase

The initial phase focuses on partitioning the majority class to achieve an ideal ratio relative to the minority class distribution.

- Cluster Count (*K*): The number of clusters *K* is set equal to the cardinality of the minority class $|B^{-}|$.
- Clustering Process: The algorithm applies Convex-Hull-Clustering to the cleaned majority set $B_{clean}^{+}$.
- Geometric Constraint: It is maintained that the convex hull of each cluster $C_{k}$ does not encapsulate any minority samples from $B^{-}$.

### 3.5. Phase 2: Ensemble Construction

The second phase focuses on building an ensemble of classifiers through iterative balanced sampling and collective decision-making.

- Iterative Sampling: For each iteration r=1,…,R, a balanced subset $A_{r}$ is formed. This subset consists of the entire minority set $B^{-}$ and a representative set of majority samples. The latter is constructed by randomly selecting exactly one sample from each of the *K* majority clusters, thereby forming a balanced subset $A_{r}$ that consists of the entire minority set $B^{-}$ and *K* representative majority samples.
- Training: A base classifier $f_{r}$ is trained on each resulting balanced subset $A_{r}$.

To classify a new observation x, the framework employs a majority voting rule across the ensemble:

(1) $$F\left(x\right)=\mathrm{sign}\left(\sum_{r=1}^{R}f_{r}\left(x\right)\right)$$

where the decision logic is defined as:

(2) sign(z)=+1ifz≥0,else−1

This voting mechanism ensures a positive final prediction if the sum of individual classifier outputs is positive and negative otherwise.

### 3.6. TPC+ Framework Implementation

The proposed TPC+ framework integrates overlap cleaning and class-imbalance handling into a unified two-phase process to improve data quality in classification tasks. By combining these two mechanisms, the framework seeks to produce a dataset with reduced class overlap and a more balanced class distribution, thereby enabling classifiers to better distinguish between minority and majority classes.

The framework begins with an overlap-cleaning stage. Algorithm 1 illustrates the overall workflow of the TPC+ framework. In its implementation, ENN is applied with the parameter *k* = 3, meaning that each majority instance is evaluated based on its three closest neighbors. The selection of k = 3 for the ENN is deliberate, serving as the optimal balance for overlap reduction. We avoided k = 1 because it lacks a consensus mechanism and is overly sensitive to isolated noise. Conversely, using larger values allows the majority class to dominate local regions, causing the unintended deletion of informative instances near the decision boundary [19]. This process produces a refined dataset with reduced class overlap, resulting in clearer class boundaries prior to the CI handling stage.

After overlap cleaning, the method proceeds to the first phase of CI handling. At this stage, the data are separated into minority ($B^{-}$) and majority ($B^{+}$) classes. The number of clusters *K* is set equal to the total number of minority samples. The majority instances are then partitioned into *K* clusters using Convex Hull Clustering, the results of which are fed directly into the second phase.

The second phase of the method focuses on constructing an ensemble of classifiers based on the clustering of majority samples obtained in the first phase. Assuming the majority class has been partitioned into *K* distinct clusters, the algorithm generates *R* balanced training datasets. In each iteration, a balanced subset is formed by retaining the entire set of minority samples and combining them with a subset of majority samples. This majority subset is constructed by randomly selecting exactly one representative sample from each of the *K* clusters. Subsequently, a specific base classifier is trained on each of these *R* balanced dataset. To classify a new observation, the algorithm employs a majority voting rule across the ensemble. If the total sum of the predictions from the *R* classifiers yields a non-negative value, the instance is assigned to class +1; otherwise, it is assigned to class −1. For the implementation of the proposed method, the number of base classifiers is set to R=5. This odd number effectively breaks any potential ties during the majority voting process. Regarding the parameter R=5, we conducted a preliminary empirical evaluation using candidate values of 3, 5, 7, 10 across a subset of the datasets. The results demonstrated that R=5 consistently provided the most optimal performance. Consequently, this value was adopted and uniformly applied to all datasets evaluated in this study.
**Algorithm 1:** TPC+
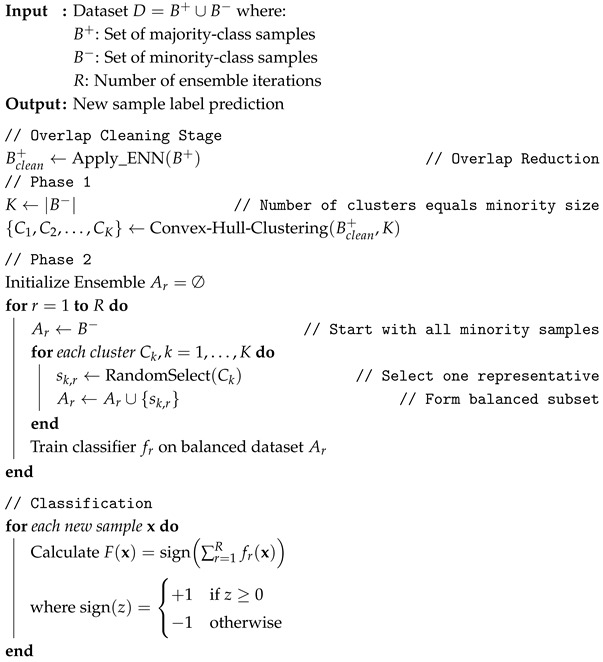



## 4. Experiment Setup

### 4.1. Baseline Methods and Parameter Settings

In the conducted experiments, various sampling methods such as RUS [22], ROS [15], Tomek links [23], ENN [19], NCR [24], KMSMOTE [25], CTGAN [26] were employed to compare the performance against the proposed method. In this study, the No Sampling condition is used without resampling, while the standard TPC [17] method is also included to assess performance differences and the improvements achieved by the proposed approach. In addition, several popular classifiers were used for customer churn prediction [30], namely Decision Tree, Logistic Regression, Random Forest, K-Nearest Neighbors (K-NN), and ensemble learning methods, that is, CatBoost, Extreme Gradient Boosting (XGBoost), and Gradient Boosting Machine (GBM).

Parameters for all classifiers were tuned using the travel industry dataset, which serves as a representative case of moderate-sized data featuring significant CO. The resulting configurations were then applied consistently across all datasets. This setup was intentionally chosen to maintain a focused evaluation of the preprocessing stage. By fixing the classifier configurations, we minimize the influence of model-specific optimization, thereby ensuring that the performance variations observed are primarily attributable to the proposed overlap reduction and balancing framework. This approach provides a more transparent assessment of how the data distribution affects the overall classification outcome [31]. The experimental parameters are summarized in Table 1. To ensure the reliability and robustness of the results, a stratified 5-fold cross-validation strategy was adopted for training and testing, providing a more stable and unbiased evaluation of the ML-SS performance.

### 4.2. Profile of Datasets

The datasets used in this study originate from Iranian telecom https://archive.ics.uci.edu/dataset/563/iranian+churn+dataset (accessed on 20 July 2025), telecom https://github.com/mahayasa/gan-hybrid-sampling-customer-churn (accessed on 22 July 2025), banking https://www.kaggle.com/datasets/adammaus/predicting-churn-for-bank-customers (accessed on 23 July 2025), online retail https://archive.ics.uci.edu/dataset/502/online+retail+ii (accessed on 23 July 2025), and travel industry https://github.com/JTarakRam/Tour-Travels-Customer-Churn-Prediction (accessed on 30 July 2025). All datasets are imbalanced with moderate ratios.

Table 2 presents the profile of the datasets used in this work. IR refers to the imbalance ratio, which represents the proportion between the positive and negative classes. Samples refers to the dataset size, while the OVC (Overlap Coefficient) represents the initial degree of overlap within each dataset.

Several approaches for identifying class overlap have been proposed in recent years; however, none have been generally applicable [32]. This is primarily due to their high sensitivity to complex data geometries and varying local densities, which can lead to inconsistent interpretations of the decision boundary across different datasets. In this study, the percentage of class overlap is estimated based on the number of instances misclassified by the kNN algorithm [32]. The formula used to estimate the overlap is presented in Equation (3). In this context, a region refers to the geometrical boundaries within the feature space that encompass a specific set of data points. Specifically, the minority class region is defined as the rectangular area that encapsulates all minority samples, while the overlapping region denotes the portion of this minority area that is also occupied by majority class samples.

(3) $$\mathrm{Overlap}\;\mathrm{Coefficient}\;(\%)=\frac{\mathrm{Instances}\;\mathrm{in}\;\mathrm{overlapped}\;\mathrm{area}}{\mathrm{Instances}\;\mathrm{in}\;\mathrm{minority}\;\mathrm{area}}\times 100$$

### 4.3. Data Preprocessing

Within each cross-validation split, a preprocessing step was performed on the training fold to scale the features before any sampling methods were applied, ensuring that the test fold remained strictly unseen. This process involved data standardization using StandardScaler. Standardization reduces value distortion, equalizes feature scales, and preserves statistical interpretability. As a result, all features were transformed to have a mean of 0 and a standard deviation of 1, ensuring consistent scaling across features [33]. The formula used to calculate the standardization is presented in Equation (4). In this study, *Z* denotes the standardized value, and *x* represents the original value of a specific feature. The parameters μ and σ correspond to the mean and standard deviation of the dataset, respectively.

(4) $$Z=\frac{x-\mu }{\sigma }$$

### 4.4. Evaluation Metric

The evaluation metric used in this study is the Geometric Mean (G-mean). The G-Mean evaluates the equilibrium between sensitivity and specificity, providing a comprehensive assessment of the classifier performance across both positive and negative classes, which is crucial for addressing CI. A higher G-mean value indicates a more equitable and balanced performance of the algorithm in distinguishing between the two classes [34]. The formula used to calculate the G-mean is presented in Equation (7).

Sensitivity measures the ability of the classifier to correctly identify positive instances.

(5) $$Sensitivity=\frac{TP}{TP+FN}$$

True Positive (TP) denotes instances in which the model correctly identifies the positive class, while False Negative (FN) refers to cases in which the model fails to detect a positive sample, incorrectly classifying it as negative.

Specificity measures the ability of the classifier to correctly identify negative instances.

(6) $$Specificity=\frac{TN}{TN+FP}$$

True Negative (TN) represents cases where the model accurately recognizes the negative class, while False Positive (FP) corresponds to instances in which the model incorrectly predicts a negative sample as positive.

By utilizing the components above, the G-Mean is calculated as the square root of the product of sensitivity and specificity.

(7) $$G\text{-}Mean=\sqrt{Sensitivity\times Specificity}$$

## 5. Results and Discussion

### 5.1. Impact of Sampling Methods on Soft Sensor Performance

We evaluated TPC+ on various imbalanced datasets characterized by different imbalance ratios and overlapping degrees. Overall, the experimental results reveal a complex dynamic between the quantity of data retained and the quality of the feature space generated by each sampling method. This evaluation highlights several key scenarios and phenomena as follows.

#### 5.1.1. Comparative Analysis of TPC+ and Standard TPC

As shown in Table 3 provides a comprehensive comparison of G-mean values across various sampling methods and classifiers applied to five service industry datasets.

Across nearly all classifier scenarios and datasets, our proposed method TPC+ consistently delivers G-mean values that are equal to, or higher than, the baseline method Standard TPC. The most notable improvement is observed in ensemble algorithms for datasets like Banking (e.g., an increase from 0.74 to 0.76 for both Random Forest and XGBoost).

This boost proves that the additional mechanisms in TPC+ effectively handle CO and facilitate a better clustering process. While standard TPC focuses on class balancing through clustering, TPC+ actively identifies and cleans ambiguous samples at the decision boundaries by strictly targeting only the majority class. By restricting the data-cleaning process to majority instances, removing this overlap reduces cognitive noise for the classifier, allowing for more precise model building without sacrificing the topological structure or the recognition rate of minority classes. This also directly addresses the existing research gap, where the standard TPC faces challenges on datasets with significant CO, such as banking and telecom. The methodological advancements integrated into TPC+ enable it to outperform the majority of classifiers on these two datasets, demonstrating its capability in handling complex data distributions.

However, in scenarios involving datasets with low overlap, such as iranian telecom, TPC+ and standard TPC demonstrate equivalent performance. This indicates that the overlap removal phase in TPC+ yields marginal impact when the class boundaries are already well-separated. Similarly, in the case of the online retail dataset, which presents moderate levels of imbalance and overlap, the performance remains comparable. These results suggest that when the initial class separation is sufficient, the additional cleaning process does not provide a significant enough boost to further enhance the classification capabilities.

#### 5.1.2. The Dominance of ROS and RUS

Conventional sampling methods like ROS and RUS show strong dominance, frequently ranking among the top performers in terms of G-mean, particularly when paired with bagging and boosting algorithms such as RF, GBM, CatBoost, and XGBoost. This phenomenon highlights a fundamental trade-off between data quality and data abundance. Complex ensemble algorithms require massive data volumes to build thousands of diverse tree splits. ROS caters to this by duplicating minority data without removing any majority samples, providing the ensemble with the full structural information needed for a stable model. Similarly, while RUS discards data, its random nature often preserves extreme borderline points that these models exploit to artificially maximize sensitivity. This approach to balancing class prior probabilities tends to improve the recall of the minority class, which is an important component of the G-mean metric. Furthermore, the robust internal regularization of modern classifiers like CatBoost and XGBoost effectively mitigates the typical overfitting risks associated with ROS.

In contrast, TPC+ performs smart undersampling that significantly improves class separation and provides a cleaner decision boundary. However, this process inherently reduces the total number of available samples. Consequently, the raw power of ROS and RUS in forcing class parity remains highly effective in scenarios where the global data structure is relatively straightforward, whereas the data reduction in TPC+ can occasionally lead to local underfitting in highly complex algorithms.

#### 5.1.3. Topological Complexity in the Banking Dataset

The banking dataset consistently yielded the lowest G-mean values (ranging from 0.41 to 0.77) across all methods, making it the most challenging dataset in this study. This global performance dip suggests that the banking dataset suffers not just from class imbalance, but also from a dense, non-linear distribution (severe intra-class overlap). In linear scenarios, such as Logistic Regression (LR), TPC+ (0.68) slightly trails RUS (0.69) because TPC+ extracts complex topological clusters that purely linear models fail to utilize. However, the true strength of TPC+ is revealed when paired with tree-based models. On the Decision Tree classifier, TPC+ generally outperforms the other methods, as its overlap-reduction phase allows the algorithm to find more precise and less noisy split points. Furthermore, when tested against high-performance boosting models like CatBoost and GBM, TPC+ maintains a competitive edge by achieving performance levels statistically equivalent to the best-performing methods. This underscores that while the banking dataset is inherently difficult, TPC+ provides a robust data foundation that enables both traditional and advanced non-linear classifiers to reach their maximum potential.

#### 5.1.4. Limitations of Minimalist Preprocessing

The absolute baseline (No Sampling) consistently produced the lowest G-mean values, particularly in parametric models like Logistic Regression (e.g., 0.41 on the banking dataset). Interestingly, using Tomek Links often provided only marginal improvements or was even identical to No Sampling (as seen with Decision Trees on the iranian telecom and banking datasets). The poor performance of No Sampling confirms the standard algorithmic bias: favoring global accuracy at the expense of the minority class, which immediately tanks the G-mean score. Meanwhile, Tomek Links is a very conservative undersampling method, it only removes the closest pairs of overlapping data between classes. In datasets with extreme imbalance and vast overlap regions, removing a few borderline samples is simply not enough to shift the decision boundary significantly.

#### 5.1.5. Impact of ENN, NCR and CTGAN

Heuristic undersampling methods like ENN and NCR generally performed better than No Sampling and Tomek Links, but they rarely reached the top spots and often lagged behind TPC+. ENN and NCR work by eliminating majority class samples misclassified by their nearest neighbors. This is quite effective at cleaning the local sample space and sharpening the boundaries, which explains why they outperform Tomek Links. However, the fundamental flaw in these heuristic methods is the lack of a global balancing mechanism. While the borderline areas become cleaner, the macro imbalance ratio often remains unchanged. Consequently, the classifier still struggles to generalize minority patterns optimally compared to methods that target the global scope like TPC+ or ROS/RUS.

The application of CTGAN across the tested datasets demonstrates a lack of competitive edge, as its G-mean scores consistently trail behind both traditional resampling techniques and modern alternatives like TPC+. While CTGAN occasionally offers marginal improvements over the imbalanced baseline in linear models like LR, it frequently degrades the performance of robust ensemble classifiers such as RF, GBM, and XGB, often recording the lowest scores in their respective categories. These results suggest that, within this experimental framework, GAN-based data synthesis fails to match the reliability and effectiveness of simpler methods like ROS or specialized sampling approaches, which dominate the top performance tiers across all classifiers.

#### 5.1.6. Inconsistency of Hybrid Methods

Hybrid methods like KMSmote which conceptually resembles TPC as both leverage clustering showed inconsistent performance. In some scenarios (like KNN on online retail), it scored very high (0.91), but on complex datasets like banking, performance plummeted (e.g., 0.57 on Logistic Regression, well below ROS at 0.69). KMSmote is designed to cluster the minority class using K-Means to find safe areas before generating synthetic samples SMOTE, aiming to avoid creating noise. The failure of KMSmote on certain datasets reveals the sensitivity of K-Means to dense, non-linear feature spaces. If the minority distribution is highly scattered or heavily overlaps with the majority, K-Means fails to define representative clusters. This can lead to overgeneralizing samples in only specific sub-regions, ignoring more difficult minority areas. This is where the comparative advantage lies, instead of immediately adding data based on clusters that might be biased, TPC+ prioritizes actively resolving the overlap issue first. By ensuring the cluster space is clear of ambiguity before balancing class weights, TPC+ offers much higher performance stability across various levels of data complexity.

### 5.2. Evaluation of Class Overlap

In evaluating class overlap reduction, the overlap percentage is computed as the ratio of the number of minority class instances in the overlapping region to the total number of minority class instances, as in Equation (3). To assess the effectiveness of the methods, we measured the overlap reduction rate, defined as the percentage reduction in overlap between the before and after states. The overlap coefficient will decrease if data cleaning increases the local density of minority class members. Conversely, the overlap coefficient can increase if the undersampling of the majority class forces k-NN to search for neighbors in more distant regions. The initial overlap values are shown in Table 2, while Table 4 shows the percentage reduction rate.

The analysis of overlap reduction effectiveness reveals significant disparities in the ability of each method to clean the feature space. TPC+ consistently demonstrates outperforms performance compared to its baseline, standard TPC, with a notable improvement in the travel industry dataset (increasing from 57.09% to 71.39%). This proves that the cleaning mechanism in TPC+ is far more effective at defining class boundaries than a single clustering approach. In contrast, traditional methods such as Tomek Links and NCR exhibit highly limited performance, nearly failing on the iranian telecom dataset with a reduction rate of only 1.78%. This confirms that pair-removal-based methods are too conservative to handle massive data overlap, as they only eliminate samples at the immediate boundaries without addressing the deep overlap density within the distributions. Meanwhile, filtering methods like ENN yield moderate but unstable results, frequently lagging behind TPC+ due to their heavy reliance on static *k*-value selection. Distinct from elimination-based methods, ROS and CTGAN record exceptionally high reduction rates, reaching up to 98% in the telecom dataset.

However, these figures must be interpreted critically, overlap reduction in oversampling based methods is often artificial, resulting from the replication or synthesis of minority samples that forcibly narrow empty spaces, which in turn increases the risk of overfitting. Conversely, RUS achieves a reasonable balance with moderate reduction rates but fails to match the precision of TPC+ in preserving data representativeness. Overall, the ability of TPC+ to achieve high reduction rates through real data cleaning rather than synthetic manipulation positions it as the most reliable solution for creating a more learner-friendly data distribution for classifiers. Nevertheless, it should be acknowledged that for certain classifiers, sampling outcomes with reduced data diversity may not yield the maximum optimal effect.

Specifically, the Tomek Links method demonstrates that removing majority-class instances forces the *k*-NN algorithm to search for more distant neighbors in the feature space to satisfy the *k*-value criterion.

Overlap detection utilities based on the *k*-nearest-neighbor (*k*-NN) approach are highly sensitive to local density, feature scaling, and parameter selection. Consequently, a minority instance may still be classified as overlapped if the new, more distant neighbor belongs to the majority class, even though the actual distance between the classes has increased and class separability has improved. This circumstance is seen as a limitation of the class overlap measurement approach, which lacks universal applicability [32]. These limitations open up new opportunities for further development of adaptive overlap detection mechanisms that utilize dynamic distance thresholds or density-aware metrics.

### 5.3. Sensitivity Analysis

**Figure 2:**
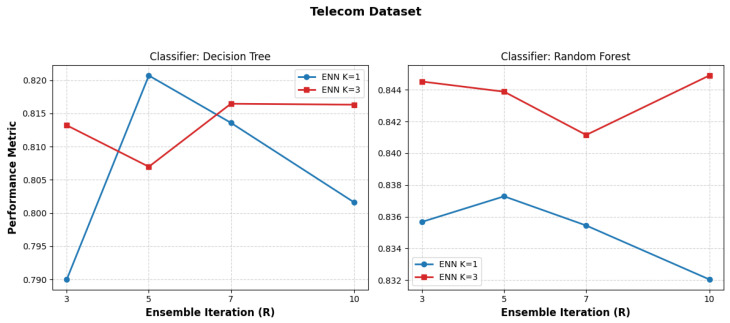
Comparison Parameter on The Telecom dataset.

**Figure 3:**
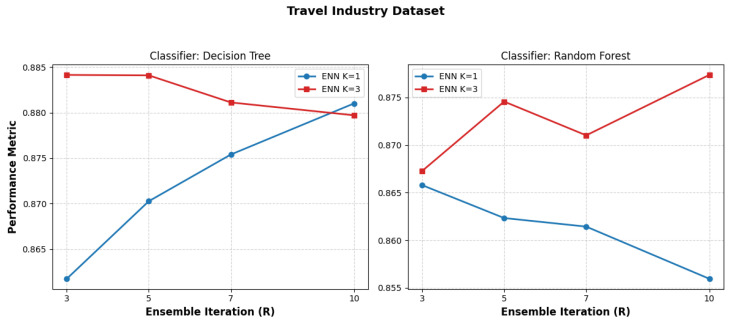
Comparison Parameter on The Travel Industry dataset.

To evaluate of the proposed methodology and justify the parameter selection as shown in Figure 2 and Figure 3, a sensitivity analysis was conducted to evaluate the effects of the number of ensemble iterations (*R*) and the number of nearest neighbors (*k*) in ENN. The evaluation utilized the travel industry and telecom datasets, which inherently feature medium-to-high CO, providing a realistic testbed for parameter stability.

In our approach, the data-cleaning phase strictly operates on the majority class to prevent the accidental removal of scarce minority samples. Consequently, the parameter *k* determines the aggressiveness of this localized cleaning. The experimental results demonstrate that k=3 generally yields a more stable performance compared to k=1, particularly when evaluated with ensemble classifiers like Random Forest. Using k=1 makes the algorithm highly sensitive to local density. This instability is clearly visible in the DT performance on the telecom dataset, where k=1 produces a sharp, erratic spike in performance at R=5 followed by a significant drop at higher iterations. By utilizing a neighborhood consensus with k=3, the algorithm avoids treating structural overlap as mere noise. This stricter removal criterion effectively reduces the overlap region while preserving the majority class distribution, leading to more predictable behavior across iterations. The parameter *R* dictates the depth of the iterative sampling process. Across both datasets, setting the iterations to R=5 emerges as a practical and efficient baseline. For robust classifiers such as Random Forest, R=5 consistently capture significant early performance gains. Beyond this point (e.g., at R=7 or R=10), the performance either plateaus, exhibits marginal improvements, or experiences slight degradation due to the potential over-cleaning of informative majority instances.

While the empirical results indicate that k=3 and R=5 serve as a robust static configuration for datasets with substantial class overlap, we acknowledge that these parameters are not universally optimal. The observed performance volatility, such as the specific isolated peak of k=1 in the telecom dataset, highlights that parameter sensitivity is inherently dataset dependent. Different data distributions and varying degrees of overlap may require fine-tuning. Moving forward, it is essential to set adaptive parameters for sampling methods according to the characteristics of the data [35].

### 5.4. Separability Analysis via Fisher’s Discriminant Ratio (FDR)

To provide a more granular understanding of how various sampling methods modify the feature space, we utilize the Fisher’s Discriminant Ratio (FDR) as a quantitative metric for class separability and class overlap [36]. While traditional performance metrics like G-mean evaluate the final classifier output, FDR offers intrinsic insights into the geometric. The FDR assesses the discriminatory power of features by comparing the distance between class centroids relative to their internal scatter. For a binary classification problem, the FDR for a given feature is defined as follows:

(8) $$FDR=\frac{{\left(\mu_{1}-\mu_{2}\right)}^{2}}{\sigma_{1}^{2}+\sigma_{2}^{2}}$$

where $\mu_{1}$ and $\mu_{2}$ denote the means (centroids) of the minority and majority classes, respectively, while $\sigma_{1}^{2}$ and $\sigma_{2}^{2}$ represent their corresponding variances.

**Figure 4:**
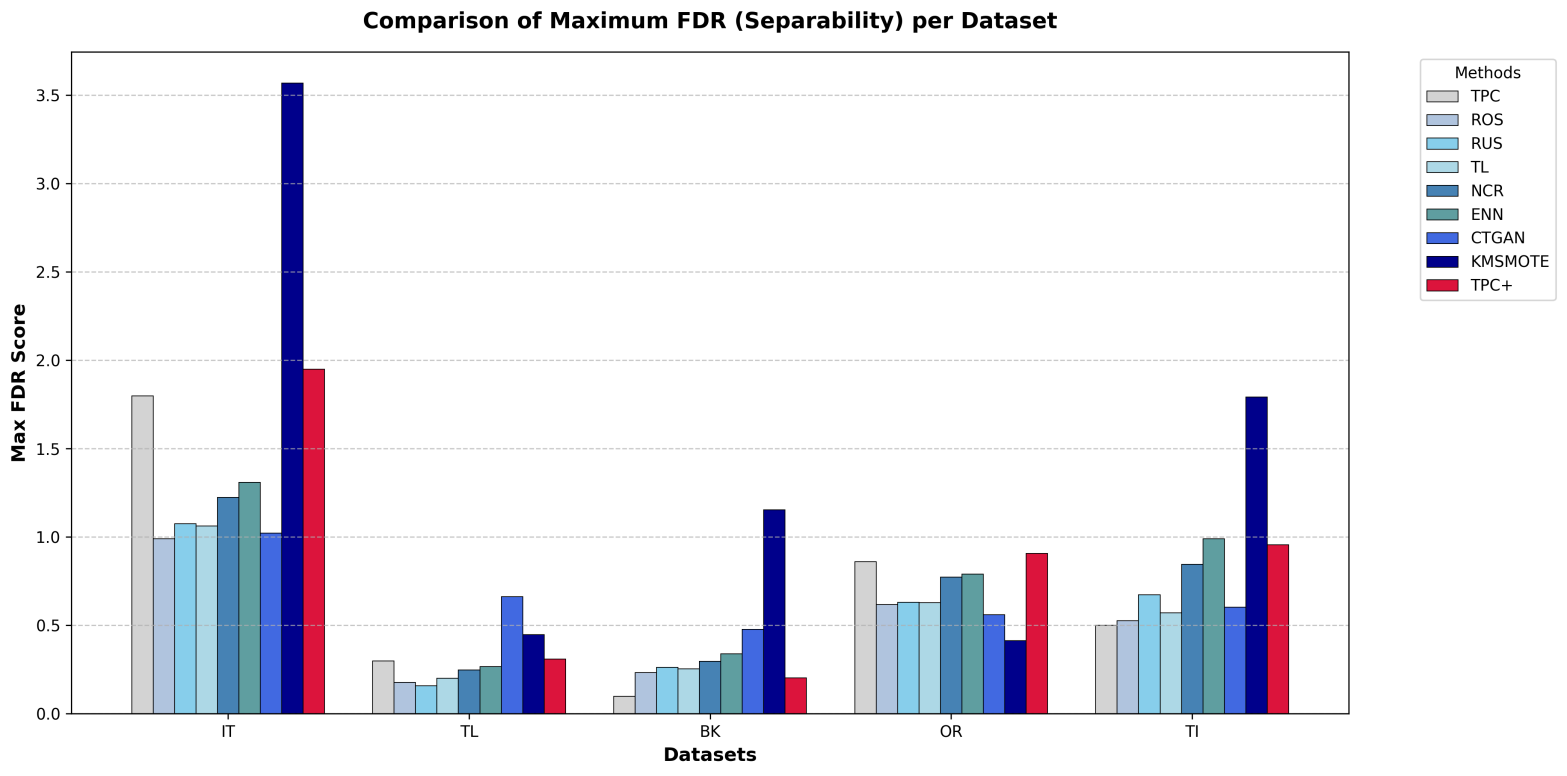
Fisher’s Discriminant Ratio.

In the context of imbalanced learning, a high FDR score indicates that a sampling method has effectively enhanced the core capabilities of the classes by either increasing the inter-class distance or suppressing the intra-class variance. As observed in our analysis (see Figure 4), methods such as TPC+ and KMSMOTE often achieve maximum FDR scores by refining the decision boundary and removing samples that contribute to class overlap.

However, a higher FDR does not always guarantee a linear increase in classification performance. A theoretical trade-off exists: while an extremely high FDR suggests a clean separation, it may also indicate a risk of over-specialization where the sampling method creates an artificially wide margin that does not represent the true underlying distribution of the data. Conversely, methods like ROS typically maintain a moderate FDR because they focus on density amplification rather than geometric repositioning. ROS preserves the original feature geometry and inherent variances, which can be advantageous for ensemble-based classifiers that rely on a more holistic representation of the class distribution to achieve better generalization.

### 5.5. Ratio After Resampling

In this section, we evaluate the data distribution following the resampling process. Table 5 presents the number of instances after resampling alongside the resulting class balance ratios. The TPC+ framework consistently achieves an ideal class distribution (1:1) across all datasets while maintaining a significantly more controlled and compact sample size compared to oversampling methods. This indicates that TPC+ effectively handles CI and reduces overlap without inflating the dataset unnecessarily. Similarly, the standard TPC method, RUS, CTGAN, and ROS also achieve a 1.0:1 ratio.

In contrast, methods such as Tomek Links, ENN, and NCR retain a relatively large number of instances but exhibit high imbalance ratios, ranging from approximately 2.6:1 to 5.9:1. This indicates a strong tendency to preserve majority class dominance, which often hinders the classifier from learning the minority class effectively. While ROS and KMSMote succeed in reaching a balanced 1:1 ratio, they do so by significantly increasing the total number of samples.

Compared with these methods, TPC+ offers a trade-off: it achieves the ideal class balance seen in ROS and RUS, but with a much higher data representativeness than RUS and is more efficient than ROS or KMSMote.

Although the final dataset size of TPC+ is comparable to that of RUS, the underlying reduction mechanisms are fundamentally different. TPC+ does not discard majority samples at random. Rather, the reduction is a direct result of the ambiguity clearing phase and the extraction of representative clusters. Consequently, this reduction is specifically designed to minimize the loss of crucial information while simultaneously maximizing the structural clarity between classes that was previously obscured by overlap. This explains why, despite having a smaller data volume, the performance of TPC+ remains competitive or even outperforms ROS in several classifiers, as evidenced by the G-mean values in Table 3.

We acknowledge that the large-scale data reduction shown in Table 5 may limit the diversity of the training data. For high-capacity modern machine learning algorithms such as deep learning or complex ensembles that thrive on data variety this shrinkage could potentially hinder the model from reaching its full generalization potential. This condition presents promising opportunities for enhancement in future framework developments. To maintain data diversity without compromising the refined feature space quality established by TPC+, data augmentation techniques or hybrid sampling strategies could be integrated.

### 5.6. t-SNE Visualization

**Figure 5:**
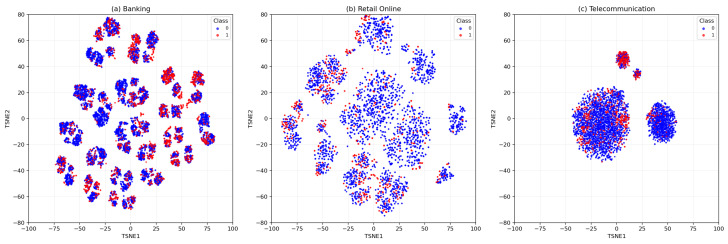
t-SNE visualization of Banking, Online Retail, and Telecom datasets before applying TPC+.

**Figure 6:**
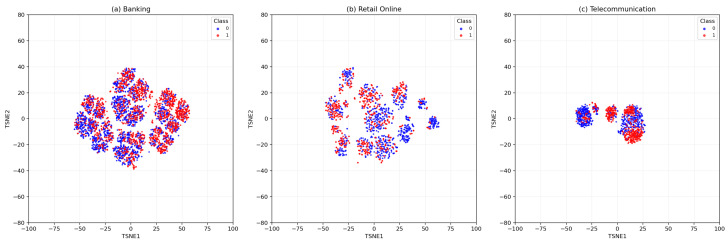
t-SNE visualization of Banking, Online Retail, and Telecom datasets after applying TPC+.

The methods used to determine overlap conditions generally involve visualizing with t-distributed stochastic neighbor embedding (t-SNE) [37]. The visualization results indicate the extent to which the sampling method reduces class overlap. This section presents a comparison of the visualizations before and after the implementation of TPC+, as shown in Figure 5 and Figure 6.

Figure 5 illustrates the distribution of the datasets used in this study, where Figure 5a represents the banking dataset, Figure 5b online retail, and Figure 5c shows the telecom dataset. Based on the t-SNE visualization, the number of instances between positive and negative classes is imbalanced, and several regions exhibit overlap between the two classes. This overlap makes it challenging to clearly distinguish whether a data point belongs to class 0 or class 1. Such overlapping conditions highlight one of the primary challenges that this study aims to address.

Figure 6 presents the visualization results after applying the proposed moderate overlap cleaning and resampling procedure to achieve class balance. A clear contrast is observed between the visualizations before and after the process: the imbalanced, overlapping distributions (Figure 5) become more balanced and distinct after sampling and cleaning (Figure 6). The resulting distributions indicate that positive and negative classes are more separable, reducing overlap while preserving the original dataset characteristics. Although a certain degree of overlap is inherent to the data structure, this moderate reduction improves the overall soft sensor performance.

### 5.7. Average Rank, Resampling Time and Standard Deviation

To evaluate the relative performance of the compared methods across various classifiers and datasets, we employed a ranking-based analysis using G-mean metrics, which focus on consistency rather than absolute values, as shown in Table 6. In terms of average rank, the closer a value is to 1, the better and more consistent the global performance; conversely, higher values signal inferior results.

ROS and RUS claim the top global spots with scores of 2.11 and 2.54, respectively. Based on the average rank interpretation, this makes them the most consistent performers in the study. However, the most critical takeaway from this global evaluation is the position of TPC+ (4.45), which convincingly outperforms its baseline, Standard TPC (5.60). This significant jump in ranking serves as the strongest statistical evidence that the extra overlap-handling mechanism proposed in TPC+ effectively improves the quality of the decision boundary compared to standard clustering approaches.

Furthermore, TPC+ successfully outranks all complex heuristic and spatial analysis methods, surpassing NCR (4.55) and ENN (4.61) and soundly beating the cluster-based oversampling method, KMSMote (6.15). This fact solidifies TPC+ as the premier complex data-level method in this comparison. The No Sampling scenario ranked worst globally (8.82), empirically validating our research premise: leaving industrial datasets imbalanced and overlapping drastically damages classifier sensitivity. Interestingly, CTGAN and Tomek Links occupied the lower performance tiers with average ranks of 8.45 and 7.67, respectively. This confirms that even advanced generative synthesis or minimalist boundary reduction is simply not enough to handle the complex data topologies found in real-world industries compared to the more robust spatial filtering and hybrid strategies.

The standard deviation values across all methods were minimal, ranging from 0.01 to 0.02. This low variance demonstrates high consistency and robustness in the classification performance across multiple independent runs.

When we synthesize these findings with execution time, TPC+ offers a truly unique value proposition. While simple heuristics like ROS/RUS are the fastest (0.01s), they do not actually refine the decision boundaries between classes. Among the family of algorithms that actively analyze and clean data geometry, TPC+ is the most optimal. Not only does TPC+ cut the computation time of Standard TPC nearly in half (from 16.615s down to 8.981s), but it simultaneously boosts global performance into the top 3.

The cluster formation process in TPC+ utilizes the HiGHS v1.15.1 linear programming (LP) solver, which can be computationally intensive as the dataset size increases. In practice, TPC+ functions exclusively as a data preprocessing step applied prior to model training. While it requires additional computation to clean and balance the training set, this process is strictly isolated from the inference stage. Consequently, the slightly higher training time is a strategic one-time investment. It produces a significantly more robust model with cleaner decision boundaries, without imposing any computational delay on the real-time systems.

### 5.8. Significance Test of Resampling Performance

In this section, we evaluate the statistical significance of the G-mean performance improvement achieved by TPC+ relative to baseline methods in addressing class overlap and class imbalance. The evaluation is conducted using the Wilcoxon signed-rank test, a nonparametric statistical test for assessing the significance of performance differences between methods [38]. Statistical significance was assessed using the Wilcoxon signed rank test with a significance level of α=0.05. The reported *p*-values reflect paired comparisons based on G-mean results of all evaluated classifiers for each dataset, rather than a single classifier. The comparison of the significance test results is summarized in Table 7. The TPC+ method is excluded from the rows, as it serves as the primary control variable for all comparisons.

Statistical analysis using the Wilcoxon Signed Rank Test reveals that the effectiveness of TPC+ in enhancing classification performance is highly context-dependent, varying across the tested datasets. When compared to standard sampling methods such as ROS and RUS, TPC+ demonstrates a significant competitive edge on the telecom and online retail datasets (p=0.02). However, it reaches statistical parity on the iranian telecom, banking, and travel datasets. This suggests that in specific domains, the mechanism of TPC+ offers stability compared to simple data replication or random sample removal. The competitive performance of ROS and RUS demonstrates their capacity to mitigate majority class dominance by balancing class proportions. While RUS achieves this through sample reduction and ROS through minority amplification, both methods successfully re-center the decision threshold. This ensures that the model can more accurately distinguish between categories, particularly in scenarios where adjusting class density is sufficient to clarify the learning space.

In comparison with cleaning-based methods namely ENN, NCR, Tomek Links, and the Standard TPC, TPC+ delivers a competitive yet varied performance. Notably, TPC+ consistently outperforms Tomek Links and the Baseline TPC on the banking and travel datasets (p≤0.08), while yielding comparable results to ENN and NCR across most other datasets. This confirms that while TPC+ shares functional similarities with cleaning methods in addressing class overlap, it retains a distinct advantage in handling the specific data structures found in the banking and travel sectors. Compared to CTGAN, TPC+ exhibits a statistically significant performance advantage in the iranian telecom, banking, and travel industry datasets (p=0.02), while achieving comparable results in the remaining sectors. Finally, when evaluated against the advanced hybrid approach, KMSMote, TPC+ proves to be statistically significant in three out of five datasets: telecom, banking, and travel (p≤0.05). Conversely, on the iranian telecom and online retail datasets, TPC+ and KMSMote produce statistically identical performance (p=0.47 and p=1.00, respectively). These findings solidify TPC+ as a robust alternative for datasets characterized by high class overlap complexity.

### 5.9. Ablation Study

To validate the structural contributions of the proposed TPC+ framework, a short ablation study was conducted. We isolated the primary components by comparing the full TPC+ architecture against its foundational elements: Standard TPC (representing purely imbalance handling) and ENN (representing purely class overlap reduction).

Evaluating the baseline performance using the average G-mean metric reveals a clear empirical advantage for the integrated approach. TPC+ achieved the highest average G-mean score of 0.8210, outperforming both the Standard TPC (0.8093) and the isolated ENN module (0.8044). This hierarchy is further corroborated by the Friedman test’s average ranking, where TPC+ secures the best global rank (4.45) compared to ENN (4.61) and Standard TPC (5.60). This initial evidence strongly indicates that addressing imbalance or overlap in isolation is sub-optimal for complex datasets.

Furthermore, statistical evaluation validates the necessity of this integrated approach. On highly complex datasets such as banking, TPC+ demonstrated statistically significant improvements over both Standard TPC and ENN. These findings indicate that the proposed integration helps maintain a better balance between overlap reduction and structural representation, thereby improving the overall quality of the decision boundary.

## 6. Limitations and Future Directions

While the TPC+ framework demonstrates significant efficacy in addressing CI and CO, several limitations related to its implementation, scalability, and theoretical trade-offs should be acknowledged. First, the performance of TPC+ is closely dependent on its parameter configuration. Although this setting was empirically optimized for the datasets used in this study, the optimal number of iterations may vary depending on the density characteristics and distribution complexity of the majority class. In addition, the distance threshold selection during the initial clustering phase remains a sensitive factor that requires careful tuning to prevent excessive data reduction. Therefore, developing adaptive parameter selection mechanisms represents an important direction for future research.

Beyond parameter sensitivity, computational scalability also remains a practical concern. A primary bottleneck arises from the utilization of LP solvers during the clustering phase. As both dataset size and dimensionality increase, the convergence time of the LP solver grows significantly. Consequently, while the proposed framework performs effectively on the evaluated datasets, future work should explore more scalable optimization strategies, approximation techniques, or lightweight centroid generation methods to improve computational efficiency across large-scale datasets. Another important limitation lies in the design choice of the cleaning mechanism. To preserve the integrity of rare minority instances, the cleaning process in TPC+ is intentionally restricted to the majority class. Although this strategy effectively avoids the removal of informative minority samples, it may reduce the framework’s capability to resolve overlap in cases where the minority class itself contains substantial noise or outliers. This trade-off highlights the challenge of balancing minority preservation and boundary purification simultaneously.

Furthermore, the FDR analysis indicates that TPC+ primarily emphasizes maximizing class separability and reinforcing discriminative decision boundaries. While this behavior contributes positively to classification performance in many datasets, it may also lead to over-specialized boundaries when overlap is inherently embedded within the underlying data distribution. In such scenarios, boundary refinement can occasionally produce performance statistically comparable to simpler density-preserving approaches, such as ROS, which maintain a larger portion of the original variance structure.

Finally, the current implementation of TPC+ operates as a multi-stage preprocessing framework rather than a fully unified end-to-end learning architecture. As a result, clustering, refinement, and classification are optimized independently through a sequential pipeline. This decoupled design may lead to locally optimal solutions that are not fully aligned with the classifier’s global objective function.

## 7. Conclusions

This study developed a framework, TPC+, that addresses two major challenges: overlap and imbalance. The ENN component operates by reducing overlap, yielding cleaner, more reliable training data prior to the cluster-based sampling stage in TPC.

The experimental results demonstrate that the proposed TPC+ framework consistently outperforms traditional undersampling and cleaning-based approaches. While simpler methods like ROS and RUS remain competitive in straightforward scenarios, TPC+ demonstrates enhanced robustness in handling the most challenging datasets, such as the banking dataset, which is characterized by severe class overlap and dense, non-linear distributions.

Despite its advantages, TPC+ requires additional computational effort during the training phase compared to simple random sampling methods, as it performs iterative clustering and cleaning. Furthermore, while the current mechanism significantly enhances performance in high-overlap environments, identifying the optimal boundary in extremely dense data remains a challenge. If the cleaning process is overly aggressive, it may risk altering local data densities, which suggests that further refinements in adaptive overlap identification could further improve the generalizability of the classifiers across diverse data structures.

Future research will focus on expanding the proposed framework by developing a scalable implementation of a multi-stage filtering mechanism, equipped with adaptive overlap detection and dynamic parameter tuning, to proactively identify overlapping samples and dynamically strengthen the decision boundary.

## Figures and Tables

**Table 1:** Classifier and Parameter Settings.

Classifiers	Parameter Settings
Decision Tree	max_depth = 10 min_samples_split = 10
Logistic Regression	C = 10 penalty = l2 solver = lbfgs max_iter = 10,000
K-Nearest Neighbor	n_neighbors = 5 weights = distance
Random Forest	max_depth = 10 n_estimators = 200
CatBoost	depth = 4 iterations = 300 learning_rate = 0.1 verbose = 0
Gradient Boosting Machine	learning_rate = 0.01 max_depth = 5 n_estimators = 300
Extreme Gradient Boosting	learning_rate = 0.1 max_depth = 3 n_estimators = 300 eval_metric = logloss

**Table 2:** Profile of the Datasets Used in the Experiment.

Dataset	IR	Samples	OVC (%)
Iranian Telecom	5.36:1	3150	12.32
Banking	3.91:1	10,000	48.99
Travel Industry	3.26:1	954	31.25
Telecom	6.11:1	4250	50.67
Online Retail	4.94:1	5630	38.50

**Table 3:** G-mean comparison across sampling methods and classifiers.

Classifiers	Datasets	None	RUS	ROS	TL	ENN	NCR	KMS	CTGAN	TPC	TPC+
DT	Iranian Telecom	0.86	0.89	0.89	0.86	0.88	0.87	0.86	0.82	**0.90**	**0.90**
	Telecom	0.83	0.84	0.83	0.83	0.84	**0.85**	0.83	0.82	0.82	0.81
	Banking	0.67	0.72	0.72	0.68	0.68	0.73	0.68	0.64	0.70	**0.74**
	Online Retail	0.79	0.82	**0.86**	0.79	0.83	0.82	0.82	0.77	0.82	0.82
	Travel Industry	0.84	0.87	0.87	0.84	**0.88**	**0.88**	0.81	0.80	0.87	**0.88**
LR	Iranian Telecom	0.65	**0.86**	**0.86**	0.66	0.78	0.77	0.77	0.80	0.85	0.85
	Telecom	0.45	0.76	**0.77**	0.47	0.57	0.56	0.72	0.54	0.72	0.74
	Banking	0.41	**0.69**	**0.69**	0.46	0.46	0.58	0.57	0.64	0.65	0.68
	Online Retail	0.64	0.78	**0.79**	0.64	0.72	0.78	0.78	0.70	0.77	0.77
	Travel Industry	0.65	**0.77**	0.76	0.66	0.74	0.74	0.73	0.72	0.76	0.76
K-NN	Iranian Telecom	0.89	**0.93**	**0.93**	0.90	0.92	0.92	0.91	0.89	0.90	0.90
	Telecom	0.55	**0.80**	0.76	0.59	0.67	0.71	0.77	0.55	0.57	0.62
	Banking	0.61	**0.72**	0.70	0.64	0.64	0.70	0.66	0.69	0.66	0.70
	Online Retail	0.85	0.86	**0.93**	0.85	0.86	0.91	0.91	0.88	0.74	0.75
	Travel Industry	0.79	0.83	0.81	0.78	**0.85**	0.84	0.80	0.79	0.82	0.83
RF	Iranian Telecom	0.90	0.93	**0.94**	0.90	0.92	0.92	0.92	0.86	0.90	0.90
	Telecom	0.84	**0.88**	0.87	0.85	0.87	0.87	0.85	0.78	0.82	0.84
	Banking	0.64	**0.77**	0.76	0.66	0.66	0.73	0.66	0.62	0.74	0.76
	Online Retail	0.85	0.90	**0.91**	0.85	0.86	0.87	0.87	0.77	0.86	0.86
	Travel Industry	0.81	0.86	0.85	0.81	0.87	0.86	0.83	0.80	0.86	**0.87**
CB	Iranian Telecom	0.91	0.94	**0.95**	0.92	0.93	0.93	0.92	0.90	0.92	0.92
	Telecom	0.88	0.88	**0.89**	0.88	0.88	**0.89**	0.88	0.87	0.78	0.80
	Banking	0.68	**0.77**	**0.77**	0.71	0.71	0.76	0.70	0.68	0.74	**0.77**
	Online Retail	0.85	0.90	**0.92**	0.85	0.86	0.86	0.86	0.84	0.87	0.88
	Travel Industry	0.83	0.88	0.88	0.84	0.89	0.88	0.84	0.83	0.89	**0.90**
GBM	Iranian Telecom	0.86	**0.91**	**0.91**	0.87	0.89	0.87	0.88	0.85	**0.91**	**0.91**
	Telecom	0.86	0.87	**0.88**	0.87	0.87	0.87	**0.88**	0.82	0.86	0.86
	Banking	0.64	**0.77**	**0.77**	0.67	0.67	0.73	0.67	0.64	0.75	**0.77**
	Online Retail	0.78	**0.87**	**0.87**	0.78	0.81	0.83	0.83	0.74	0.84	0.84
	Travel Industry	0.80	0.89	**0.90**	0.82	0.89	0.88	0.86	0.81	0.89	0.88
XGB	Iranian Telecom	0.91	0.93	**0.94**	0.92	0.93	0.92	0.91	0.91	0.92	0.92
	Telecom	0.86	0.88	**0.88**	0.87	**0.88**	**0.88**	0.87	0.85	0.75	0.79
	Banking	0.67	**0.77**	**0.77**	0.70	0.70	0.75	0.69	0.67	0.74	0.76
	Online Retail	0.82	0.88	**0.89**	0.82	0.85	0.83	0.83	0.82	0.85	0.85
	Travel Industry	0.83	0.89	**0.90**	0.82	0.90	0.89	0.85	0.83	0.88	**0.90**

Values in bold indicate the best performance for each row.

**Table 4:** Percentage of overlap reduction.

Datasets	TPC	TPC+	RUS	ENN	ROS	TL	NCR	KMS	CTGAN
Iranian Telecom	85.63 ↑	87.66 ↑	36.85 ↑	32.14 ↑	98.21 ↑	1.78 ↑	39.20 ↑	77.27 ↑	87.74 ↑
Telecom	83.11 ↑	89.14 ↑	80.68 ↑	26.99 ↑	98.91 ↑	4.85 ↑	40.66 ↑	87.57 ↑	98.58 ↑
Banking	67.42 ↑	73.69 ↑	66.93 ↑	45.54 ↑	62.46 ↑	9.25 ↑	37.15 ↑	91.55 ↑	57.13 ↑
Online Retail	93.84 ↑	94.18 ↑	65.19 ↑	56.18 ↑	81.53 ↑	37.01 ↑	48.36 ↑	86.75 ↑	59.92 ↑
Travel Industry	57.09 ↑	71.39 ↑	49.06 ↑	59.23 ↑	71.14 ↑	5.98 ↓	47.01 ↑	81.79 ↑	76.99 ↑

Note: Upward arrows (↑) indicate overlap reduction, while downward arrows (↓) indicate an increase in overlap compared to the original data.

**Table 5:** Number of samples after resampling and class balance ratios.

Datasets	TPC	TPC+	RUS	ENN	ROS	TL	NCR	KMS	CTGAN
Iranian Telecom	792	792	792	2497	4248	2375	2394	4256	4248
	1.00:1	1.00:1	1.00:1	5.05:1	1.00:1	5.31:1	5.05:1	1.01:1	1.00:1
Telecom	958	956	956	2919	5843	3324	2914	5862	5842
	1.00:1	1.00:1	1.00:1	5.01:1	1.00:1	5.95:1	5.09:1	1.02:1	1.00:1
Online Retail	1518	1518	1516	4062	7491	4482	4271	7512	7492
	1.00:1	1.00:1	1.00:1	4.36:1	1.00:1	4.91:1	4.63:1	1.01:1	1.00:1
Banking	3258	3260	3259	6176	12740	7603	6330	12766	12742
	1.00:1	1.00:1	1.00:1	2.79:1	1.00:1	3.67:1	2.88:1	1.01:1	1.00:1
Travel Industry	404	360	358	664	1168	755	658	1180	1168
	1.00:1	1.00:1	1.00:1	2.71:1	1.00:1	3.22:1	2.68:1	1.01:1	1.00:1

**Table 6:** Performance Comparison of Resampling Methods.

Methods	Time (s)	Average Rank	Standard Deviation
ROS	0.01	2.11	0.02
RUS	0.01	2.54	0.01
TPC+	8.981	4.45	0.01
NCR	1.15	4.55	0.02
ENN	0.42	4.61	0.02
Standard TPC	16.615	5.60	0.02
KMSMote	0.97	6.15	0.02
Tomek Links	0.041	7.67	0.02
CTGAN	16.6	8.45	0.02
No Sampling	0.05	8.82	0.02

**Table 7:** Wilcoxon Signed-Rank Test Results.

Methods	Iranian Telecom	Telecom	Banking	Online Retail	Travel Industry
Standard TPC	0.58	0.05 *	0.02 *	0.11	0.08 **
ENN	0.69	0.30	0.02 *	0.58	0.58
Tomek Links	0.11	0.69	0.02 *	0.22	0.02 *
ROS	0.08 **	0.02 *	0.94	0.02 *	0.30
RUS	0.11	0.02 *	0.16	0.02 *	0.22
NCR	0.47	0.30	0.02 *	1.00	0.11
KMSMote	0.47	0.05 *	0.02 *	1.00	0.02 *
CTGAN	**0.02**	0.81	0.02 *	0.30	0.02 *

Values marked in bold with (*) indicate statistically significant differences compared to TPC+ based on the Wilcoxon signed-rank test at α=0.05. ** Indicates marginal significance at α=0.10.

## Data Availability

The data presented in this study are available in the article.

## Abbreviations

The following abbreviations are used in this manuscript:

CB	CatBoost
CO	Class Overlap
CI	Class Imbalance
CTGAN	Conditional Tabular Generative Adversarial Network
DT	Decision Tree
ENN	Edited Nearest Neighbors
FDR	Fisher’s Discriminant Ratio
FP	False Positive
FN	False Negative
GBM	Gradient Boosting Machine
G-mean	Geometric Mean
IoT	Internet of Things
K-NN	k-Nearest Neighbors
KMSMOTE	K-Means Synthetic Minority Over-sampling Technique
LR	Logistic Regression
LP	Linear Programming
ML-SSs	Machine Learning based Soft Sensors
ML	Machine Learning
NCR	Neighborhood Cleaning Rule
RF	Random Forest
ROS	Random Oversampling
RUS	Random Undersampling
SSs	Soft Sensors
TPC	Two Phase Clustering
TPC+	Two Phase Clustering Plus
TN	True Negative
TP	True Positive
XGBoost	Extreme Gradient Boosting
